# Contrast Sensitivity and Night Driving in Older People: Quantifying the Relationship Between Visual Acuity, Contrast Sensitivity, and Hazard Detection Distance in a Night-Time Driving Simulator

**DOI:** 10.3389/fnhum.2022.914459

**Published:** 2022-07-29

**Authors:** Pete R. Jones, Judith Ungewiss, Peter Eichinger, Michael Wörner, David P. Crabb, Ulrich Schiefer

**Affiliations:** ^1^Department of Optometry and Visual Sciences, School of Health Sciences, City, University of London, London, United Kingdom; ^2^Competence Center “Vision Research”, Study Course Ophthalmic Optics and Optometry, University of Applied Sciences, Aalen, Germany; ^3^Carl Zeiss Vision International GmbH, Aalen, Germany; ^4^Study Course Mechatronics, University of Applied Sciences, Aalen, Germany; ^5^Blickshift GmbH, Stuttgart, Germany; ^6^Department of Ophthalmology, University of Tübingen, Tübingen, Germany

**Keywords:** driving, night, scotopic, visual acuity, contrast sensitivity, driving simulator, glare, aging

## Abstract

**Purpose:**

(i) To assess how well contrast sensitivity (CS) predicts night-time hazard detection distance (a key component of night driving ability), in normally sighted older drivers, relative to a conventional measure of high contrast visual acuity (VA); (ii) To evaluate whether CS can be accurately quantified within a night driving simulator.

**Materials and Methods:**

Participants were 15 (five female) ophthalmologically healthy adults, aged 55–81 years. CS was measured in a driving simulator using Landolt Cs, presented under *static* or *dynamic* driving conditions, and *with* or *without* glare. In the *dynamic* driving conditions, the participant was asked to simultaneously maintain a (virtual) speed of 60 km/h on a country road. In the *with glare* conditions, two calibrated LED arrays, moved by cable robots, simulated the trajectories and luminance characteristics of the (low beam) headlights of an approaching car. For comparison, CS was also measured clinically (with and without glare) using a Optovist I instrument (Vistec Inc., Olching, Germany). Visual acuity (VA) thresholds were also assessed at high and low contrast using the Freiburg Visual Acuity Test (FrACT) under photopic conditions. As a measure of driving performance, median hazard detection distance (MHDD) was computed, in meters, across three kinds of simulated obstacles of varying contrast.

**Results:**

Contrast sensitivity and low contrast VA were both significantly associated with driving performance (both *P* < 0.01), whereas conventional high contrast acuity was not (*P* = 0.10). There was good correlation (*P* < 0.01) between CS measured in the driving simulator and a conventional clinical instrument (Optovist I). As expected, CS was shown to decrease in the presence of glare, in dynamic driving conditions, and as a function of age (all *P* < 0.01).

**Conclusion:**

Contrast sensitivity and low contrast VA predict night-time hazard detection ability in a manner that conventional high contrast VA does not. Either may therefore provide a useful metric for assessing fitness to drive at night, particularly in older individuals. CS measurements can be made within a driving simulator, and the data are in good agreement with conventional clinical methods (Optovist I).

## Introduction

Many visually normal older people have selective difficulties driving at night (i.e., under mesopic or low-photopic illumination, and/or in the presence of glare). Thus, while night driving poses additional challenges and dangers for drivers of *all* ages (Wood, 2020), drivers older than 65 years exhibit: an increased prevalence of fatal crashes at night (Mortimer and Fell, 1989), a greater degradation of steering accuracy (Owens and Tyrrell, 1999), elevated self-reports of glare from oncoming headlights (Kimlin et al., 2016), slower recovery times after experiencing glare (Collins, 1989), and poorer recognition of road signs at night (Owens et al., 2007). Accordingly, around one in three older drivers report having restricted or ceased driving at night (Lyman et al., 2001; Naumann et al., 2011).

These night driving difficulties can be traced back to normal (non-pathological) changes in the anatomy and physiology of the aging visual system. Most, if not all, ocular structures are affected detrimentally by age. Secretions from the lacrimal and meibomian glands reduce with age, resulting in a reduction in tear film volume (dry eyes), in turn leading to irregularities on the corneal surfaces that can cause light scatter and glare (Huang et al., 2002). Cells in the corneal endothelium decrease in density and regularity, leading to accumulations of fluid in the corneal stroma and a concomitant loss of corneal transparency. Degeneration of the radial dilatator muscle leads to a progressive reduction in pupil diameter with age (senile miosis), resulting in reduced levels of incoming light, with deleterious consequences for low-light activities. The crystalline lens yellows, reducing the transmission of short-wavelength light, and fluorophores and insoluble proteins aggregate within the lens, leading to veiling glare and intraocular light scatter. Collagen fibers within the vitreous humor start to degrade, resulting in floaters that can block or scatter incoming light. In terms of the neurosensory retina, although cones are often preserved, the number of rods diminishes with age (Curcio et al., 1993), with obvious consequences for night vision. While the supporting cells of the retinal pigmented epithelium (RPE) undergo several structural changes that impair their normal function (e.g., aggregation of lipofuscin granules). This leads, in particular, to a reduced rate of rhodopsin regeneration with age, leading to impaired dark adaptation, slower glare recovery, and a general difficulty seeing at night. A loss of macular pigment, and an age-related loss and/or dysfunction of retinal ganglion cells and downstream neural pathways have also been reported (Spear, 1993; Lovasik et al., 2003), each of which are also likely to further degrade the fidelity of vision. Aging is also associated with various age-related eye disease of the orbit (e.g., enophthalmos, ptosis) and retina (e.g., glaucoma, macular degeneration, diabetic retinopathy) that may further limit vision. However, pathologies are outside the scope of the present work, which instead focuses solely on visually normal older drivers.

In short, due to a variety of anatomical changes, the aging visual system struggles to operate in low light levels, impairing night driving ability in many or all older drivers (Knoblauch and Arditi, 1992). It may therefore be prudent to obtain measures of night driving ability when assessing fitness to drive in older adults. Such assessments may become imperative in coming years, due to both demographic changes [i.e., the fact that life expectancy, and therefore the number of older drivers, is increasing rapidly worldwide (Vollset et al., 2020)], compounded by the fact that dependency on private car journeys is steadily *increasing* amongst older people (Mollenkopf et al., 2002) (perhaps representing a greater need, desire, and/or capability for individual, self-determined mobility).

Crucially, however, conventional measures of vision, such as high contrast visual acuity, visual fields, or patient self-reports, tend to be relatively poor predictors of night driving ability [for a review, see Gruber et al. (2013)]. What is needed is a strong and unambiguous predictor of night driving ability.

One promising measure is contrast sensitivity (CS). Older drivers who avoid night driving often present with reduced CS (Puell et al., 2004; Brabyn et al., 2005), and age-related media opacities, which often have little-to-no effect on photopic visual acuity, have been shown clinically to result in a pronounced deterioration in CS (Anderson and Holliday, 1995). Direct evidence regarding the efficacy of CS to predict night driving ability can be found in Wood and Owens (2005), who examined 24 experienced drivers aged between 18 and 80 years old, and found that Pelli-Robson contrast sensitivity significantly predicted the percentage of road objects (signs, pedestrians, road hazards) recognized when driving around a closed road circuit at night.

To date, data linking CS to night driving aptitude remain sparse, and have come predominantly from studies of on-road driving. The present study aimed to provide convergent evidence using a night-driving simulator. If such simulators could be shown to provide comparable data, this could also have several practical ramifications for future research. Thus, while on-road studies remain the gold standard for most applications, simulators allow high-risk scenarios to be explored, provide the ability to completely standardize/manipulate key variables, and are highly convenient (e.g., allowing night-driving to be tested in the day, and irrespective of the prevailing weather conditions).

Simulating the glare from streetlights and oncoming car headlights is not trivial, however, and in the past some simulators have been criticized for given an incomplete or “two dimensional” portrayal of night driving. Therefore, in the present project an innovative new approach is introduced, in which programmable LED light arrays are moved in real time by cable robots, in order to provide a mobile glare source that accurately simulates trajectories and luminance characteristics of oncoming car headlights (see Section “Materials and Methods”). We also developed novel software to allow CS to be assessed within the simulator, either with or without the presence of glare, and either when the simulated car is stationary (“static”) or in motion (“dynamic”). By using this novel setup, we were able to directly assess the predictive value of various CS measures on night driving ability in visually healthy older adults, and contrast this with conventional clinical measures such as photopic VA.

In short, the purpose of the present work was to assess how well CS predicts night-time hazard detection ability in normally sighted older drivers, relative to a conventional measure of high contrast VA. Our hypothesis was that photopic VA is a poor predictor of night driving performance, and that naturally arising differences in night driving ability amongst older drivers would be better captured by CS. This work was further intended to evaluate whether CS can be accurately quantified within a novel night driving simulator, and to showcase an improved method of realistically simulating headlight glare using robotic LED units.

## Materials and Methods

### Participants

Participants were fifteen ophthalmologically healthy adults (five female), aged 54.6–80.6 years (mean: 68.0 years; the distribution of ages can be seen within Figure 6 of the Section “Results”).

**FIGURE 1:**
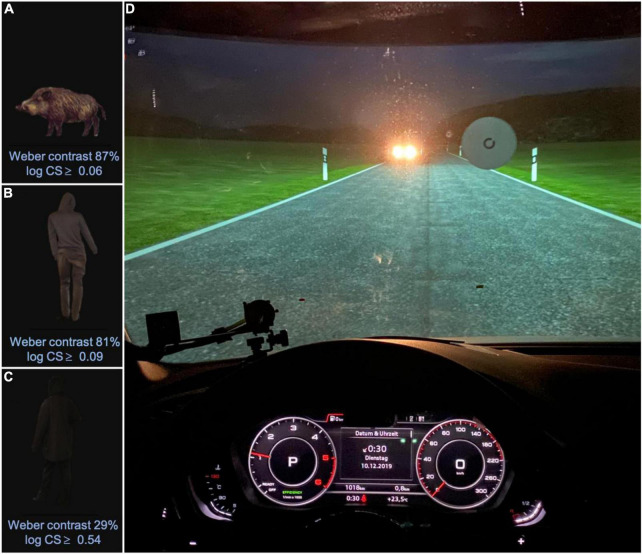
The night driving simulator **(D)** and associated hazards **(A–C)**. Hazard in Panel **(C)** may be difficult to see owing to its low contrast. Visible in Panel **(D)** is one of the Landolt Cs used to measure CS within the simulator, as well as the dipped headlights of the oncoming vehicle, which were simulated by LEDs physically moved by cable robots. The digital dashboard was fully functional and provided realistic feedback during simulated driving. Realistic auditory feedback (engine noises) was also provided. See main manuscript text for further technical details regarding the simulator. Note that when capturing the photograph shown in panel **(D)**, some overexposure was required to visualize the instrument panel and compensate for the veiling glare of the oncoming virtual car. As a result, some subtle but important aspects of the lighting may not be apparent. For example, car headlights have an asymmetrical light distribution (to minimize glare to oncoming cars). So in the scene depicted here, the righthand side of the road immediately in front of the simulated car should have a more intense luminance distribution than the left. Likewise, the forward light from the simulated vehicle should decrease with eccentricity and distance from the participant. All of these details were in fact the case, but are not obvious from inspection of the photograph (e.g., apron luminance was between 1.3 and 2 cd/m2 for the right roadside, varying as a function of distance, and between 0.6 and 1 cd/m2 for the left).

**FIGURE 2:**
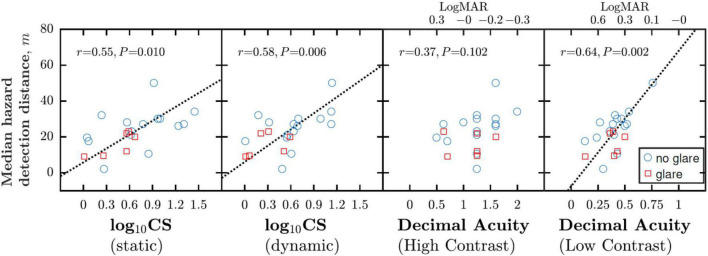
Correlations between night driving performance, as measured by MHDD, and various measures of CS (measured within the simulator) and VA (measured outside the simulator). Each marker indicates data from a single participant, with measurements made either with glare (red squares) or without glare (blue circles). Dashed lines show best fitting geometric mean (“error in both axes”) regression slopes.

**FIGURE 3:**
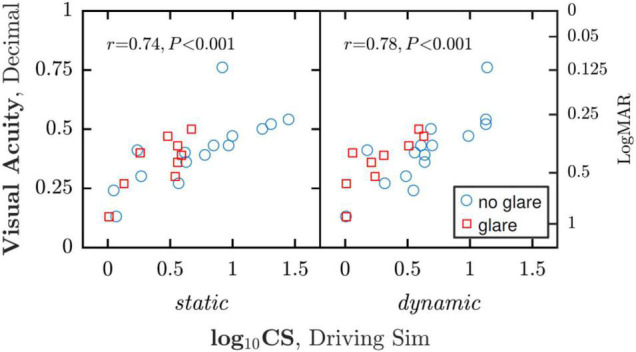
Correlations between CS (measured within the simulator) and low contrast VA (measured outside the simulator). Note that each participant contributed two data points to each analysis, and as such a more statistically correct approach would have been to use a linear mixed effects model to account for any dependencies between within-subject data points.

**FIGURE 4:**
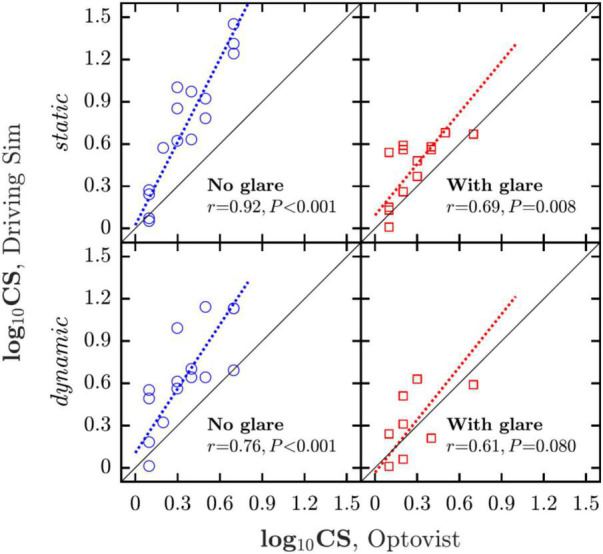
Correlations between CS measured in and out of the simulator. Dashed lines show geometric mean regression slopes.

**FIGURE 5:**
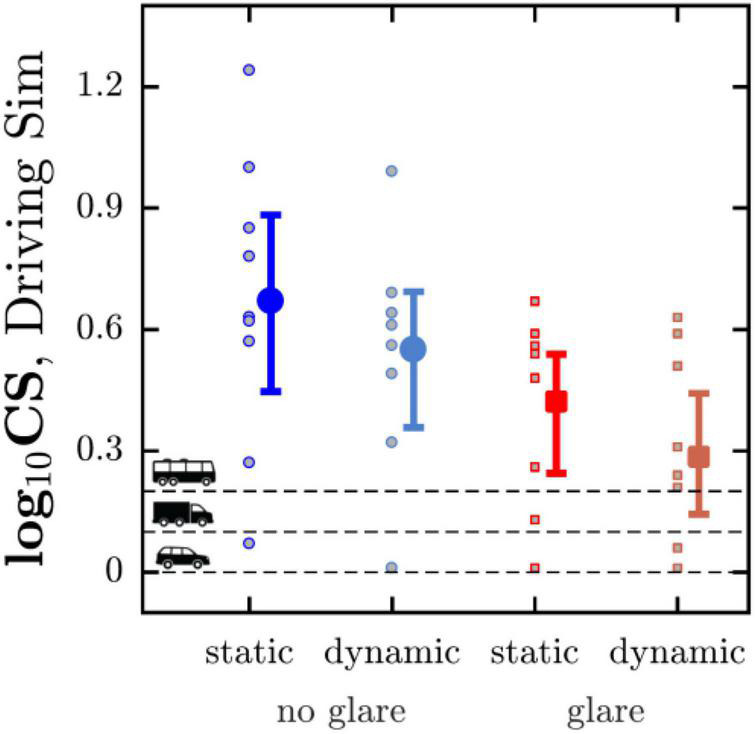
Mean (with 95% CI) CS for the four conditions assessed in the driving simulator. Horizontal dashed lines indicate the minimum CS requirements in Germany for three classes of vehicular driving licenses. Markers show the values for each individual participant. See Supplementary Figure 1 for an equivalent figure in which the four corresponding data points for each individual participant are connected by lines.

**FIGURE 6:**
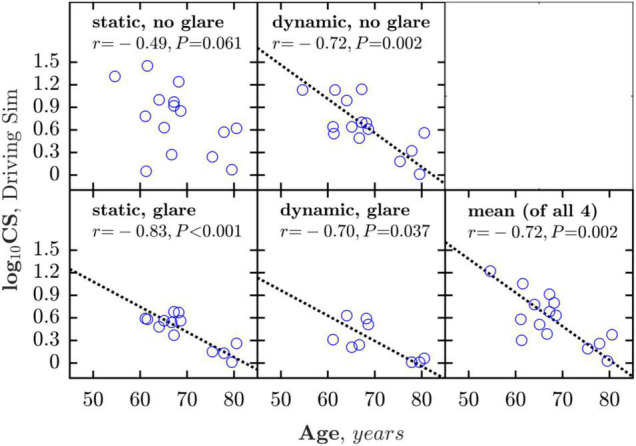
Correlations between age and CS measured under the four simulator conditions (glare/no-glare vs. static/dynamic). The bottom right panel shows mean CS averaged across all four measures. Dashed line shows geometric mean regression slope (shown only where association was statistically significant).

Ophthalmic health was confirmed by: slit lamp examination [no more than moderate lens opacity using the Lens Opacities Classification System III (Chylack et al., 1993)], refraction (maximum spherical ametropia ± 5 dpt, maximum cylindrical ametropia 2.5 dpt), static perimetry (a passing score on the Octopus 900 [HAAG-STREIT, Köniz/CH]; program FG “driver’s license,” grid 105 locations arranged from 0 to 80 degrees eccentric, strategy “2-LT,” stimulus size III, stimulus duration 200 msec, background luminance 10 cd/m^2^), visual acuity (binocular distance acuity < 0.3 LogMAR, measured under photopic illumination with habitual refractive correction), no history of ophthalmic surgery (e.g., cataract removal), and no self-reported eye disease (e.g., ophthalmic injuries or inflammations, diseases of the visual pathway, ocular motility disorders, or double vision).

Prior to the present study, all participants had also undergone a detailed ophthalmologic examination to further rule out any ophthalmic pathology. This included the assessment of ocular alignment (cover test), ocular motility (guiding movements and evaluation of saccadic velocity in horizontal and vertical direction), inspection of the anterior and middle eye segments with a slit lamp (BQ 900 LED, Haag-Streit, Köniz, Switzerland), an assessment of the chamber angle width according to the van Herrick classification (van Herick et al., 1969). And, following pharmacological mydriasis (Neosynephrin POS^®^ 5%; Ursapharm, Saarbrücken, Germany): Stereo fundus photography (WX 3D, KOWA, Düsseldorf/D), Scheimpflug documentation of the anterior eye segments using the Pentacam HR 70900 (Oculus, Dutenhofen, Germany), and non-contact (air pulse) intraocular pressure measurement using the KT-800 (Kowa, Düsseldorf, Germany).

Participants were recruited by emails circulated within the Aalen University of Applied Sciences, by local newspaper advertisements, and via ophthalmologists working in private practice. Written informed consent was obtained from all participants. The study followed the tenets of the Helsinki declaration, and was approved by the Institutional Review Board of the State Medical Association of Baden-Württemberg (F-2015-044#A2). The study protocol is registered at ClinicalTrials.gov (NCT03169855; last updated 2019-01-10).

### The Driving Simulator

#### Hardware

Figure 1 shows the participant’s view from the inside of the fixed-base night driving simulator, which was custom made at the Innovation Centre of the University of Applied Sciences, Aalen, Germany. The chassis consisted of a modified Audi A4 (Audi AG, Ingolstadt, Germany), with a digital dashboard and an integrated (contactless) eye and head tracking system (Smart Eye Pro; SmartEye, Gothenburg, Sweden). Images were projected onto a cylindric projection screen (radius: 3.2 m) by two high-performance LED planetarium projectors (VELVET LED; Zeiss AG, Jena, Germany). This set up was used to depict a night-time driving scenario, featuring a rural road and an infinite series of oncoming vehicles with dipped headlights (vehicles occurring at 990 m intervals). The headlights of the oncoming vehicle were simulated by two LED arrays, which were moved precisely along realistic trajectories by means of cable robots. The LEDs were additionally adjusted in terms of their illuminance (0.04 lx to 1.35 lux) and angle of view (−20° and −7°) in order to precisely replicate the characteristics of the low-bream headlights of an approaching VW Golf VII (Volkswagen AG, Wolfsburg, Germany).

#### Graphics

As shown in Figure 1D, optotypes with varying contrast were displayed directly to the right of the roadside, presented against a uniform gray circular background. Hazards of varying contrast (Figures 1A–C) were presented without background at the right of the roadside and disappeared when passed (see Section “Assessing Night-Time Hazard Detection Ability” for additional details regarding the hazards). To calibrate the luminance of the images displayed, measurements were performed using a spectroradiometer (CAS 140 VIS/UV; Instrument Systems GmbH, Munich, Germany) and confirmed using a spot photometer (Minolta LS160; Konica Minolta Holdings K.K., Tokyo, Japan). This confirmed that the luminance characteristics of the simulated scene corresponded, to a reasonable level of approximation (maximum deviation: 20%), to the on-road conditions of a vehicle with dimmed halogen headlights (Golf VII, Volkswagen AG).

#### Participant Seating

As with the clinical examination of mesopic contrast sensitivity (see below), each participant underwent a minimum of 15 min dark adaptation in the driving simulator, prior to any assessments. This time was used to instruct the participant about the various tasks, and to adjust the driver’s seat and the steering wheel to comfortable positions. To militate against simulator sickness, each participant was carefully instructed to avoid abrupt speed changes or steering maneuvers, and virtual driving was limited to straight lanes. In practice no such sickness was reported, though simulator sickness remains an important consideration for driving simulators in general.

### Assessing Visual Acuity

High contrast visual acuity (VA) was assessed under photopic conditions, outside of the simulator, using the Freiburg Visual Acuity and Contrast Test [FrACT (Bach, 1996)]. An adaptive BestPEST strategy was used, with 22 presentations of a Landolt C. On each trial the Landolt C was oriented in one of eight directions (“8AFC”), and the participants task was to identify the location of the gap [see Bach (2006) for details]. Stimuli were viewed binocularly, at a distance of 4 m. Low contrast VA was also assessed in the exact same manner, using low contrast Landolt C stimuli (Michelson contrast 2.5%, corresponding to logCS 1.31). This yielded one estimate of high contrast VA and one estimate of low contrast VA, per participant.

### Assessing Contrast Sensitivity

Binocular contrast sensitivity was measured both in and out of the driving simulator, and both with and without the presence of glare (2 × 2, within subjects design), as follows:

#### Assessing Contrast Sensitivity Clinically

Outside of the driving simulator, CS was assessed by the Optovist I instrument (Vistec AG, Olching, Germany), modified to allow the presentation of low contrast levels (down to logCS = 1.03). During this procedure, 8AFC Landolt Cs (VA level: 1.0 LogMAR) were presented with varying contrast until a contrast detection threshold could be determined (three out of five criterion). This process was carried out twice: once without glare (background luminance 0.032 cd/m^2^) and once with glare (background luminance 0.32 cd/m^2^; glare source: visual angle: 0.25°, eccentricity 3° to the left, corneal illumination level 0.35 lx). Note that as per the driving simulator, a dark adaptation period of 15 min was required before contrast was assessed.

#### Assessing Contrast Sensitivity Within the Driving Simulator

Within the driving simulator, CS thresholds were assessed in a psychophysically similar manner, again using 8AFC Landolt Cs (VA level: 1.0 LogMAR) that were again presented with varying contrast until a contrast detection threshold could be determined (BestPEST thresholding strategy: exactly 22 trials per run). As shown in Figure 1D, The Landolt Cs were presented directly to the right of the roadside, against a uniform gray circular background (background luminance 0.032 cd/m^2^). As with the CS measurements made outside the simulator, these measurements were again made with and without glare. In this case though the glare source was the simulated oncoming car headlights (see Figure 1D). The number of illuminated LEDs and their luminance levels were adapted to viewing angles and the previously measured lighting characteristics of an approaching vehicle (GOLF VII, Volkswagen, Wolfsburg, Germany). CS measurements were made separately under *static* and *dynamic* driving conditions, as follows. Note that here we use the term dynamic to refer to conditions in which the simulated vehicle was driving forward (as opposed to parked). The “dynamic” CS stimulus did not move, however, either locally or within the scene; rather it consisted of a stationary Landolt C, presented in the context of virtual motion.

In the *static* conditions (with and without glare), the participants’ simulated vehicle was parked at a distance of 50 m to a stationary vehicle on the opposite lane. Both vehicles remained stationary during the CS measurements.

In the *dynamic* conditions (with and without glare), the participant first accelerated the virtual vehicle to a speed of 90 km/h and was then asked to maintain this speed as constantly as able. In addition to the speedometer (with digital and pointer display), the virtual engine noise played into the passenger compartment served as an additional acoustic feedback signal for speed control. Then, after covering a distance of approximately 550 m at this speed, the participant was prompted by a road sign to reduce their speed to 60 km/h. After a distance of approximately 300 m, the 8AFC Landolt C threshold algorithm then commenced, and proceeded in the same manner as in the static condition. Participants were required to maintain driving at 60 km/h during this procedure. In summary, four CS estimates were made within the driving simulator, per participant (static + no-glare; static + glare; dynamic + no-glare; dynamic + glare). Measurements of CS without glare were always made first (static, then dynamic), followed by those with glare (static, then dynamic).

### Assessing Night-Time Hazard Detection Ability

During simulated driving participants were asked to drive along a rural road while maintaining a virtual speed of 60 km/h. At random intervals one of three hazards was presented in random order. The hazards are shown in Figures 1A–C, and consisted of a mid-contrast “wild boar” (Weber contrast = 87%), a low contrast “man dressed in gray” (Weber contrast = 81%), and a very low contrast “man dressed in black” (Weber contrast = 29%). The onset of the hazards was synchronized with the appearance of an oncoming car, which occurred at 990 m intervals. Hazards were presented against the background of the scenery and vanished immediately once passed. The participant’s task was to verbally identify the hazard. Most did so by stating the name of the object as soon as they saw it, at which point the experimenter recorded the time of response by pressing a key. Alternatively, a small minority of participants preferred to say “jetzt” (“now,” in German), and then to classify the obstacle according to the three subtypes, in which case the “jetzt” was taken as the timepoint of the response. In a small number of cases (<1%) the participant verbally corrected the obstacle type, in which case the last response was taken as the response time. No false positive responses occurred.

Note that for technical ease, and to maintain standardized levels of luminance and contrast, the three simulated hazards remained standing still, always appeared on the right side of the road, and did not change in luminance as the simulated vehicle approached. These facts made the hazards somewhat unrealistic and predictable in nature. Test runs with “no hazard presentation” were therefore interspersed to reduce anticipation.

Hazard recognition distance (HRD) was recorded as the distance of the hazard, in meters, at the time of correct identification. Each of the three stimuli were presented three times in random order, and the nine values were averaged to yield a single Median HRD (MHRD) value, per participant. In the present study, this MHRD metric was used as the index of driving performance.

### Analysis and Missing Data

De-identified data were analyzed and plotted in MATLAB 2016B (MathWorks; Natick MA, United States). 95% confidence intervals were computed using bootstrapping (*N* = 20,000; bias-corrected and accelerated method).

Not all participants successfully completed every test condition in the simulator, owing to a number of minor technical malfunctions. In particular, an error with the cable robots meant that the final five participants were unable to perform the dynamic glare condition (i.e., the LED panels had to remain fixed in place)—and due to COVID restrictions these participants were unable to return at a later data. One corollary of this is that when performing repeated measures inferential statistics, some participants who contributed only partial data had to be excluded, as detailed within the results. Note, the study findings remained qualitatively unchanged if linear mixed effects models were instead used to analyze the data (i.e., more complex statistical models that permit missing data within-subjects). These analyses are not reported, however, as they are more complicated to describe and interpret.

## Results

### Contrast Sensitivity and Visual Acuity as Predictors of Night Driving Ability

As shown in Figure 2, CS measured within the driving simulator, was significantly associated with night driving performance (MHDD), both when CS was measured in a static scene [*Pearson Correlation*; *r*_19_ = 0.55, *P* = 0.010] and in a dynamic driving scenario [*r*_19_ = 0.58, *P* = 0.006]. Conversely, the more conventional clinical measure of high contrast acuity was not correlated with MHDD [*r*_19_ = 0.37; *P* = 0.102], suggesting it is not a sensitive index of night driving performance. Notably though, low contrast acuity was correlated with MHDD [*r*_19_ = 0.64, *P* = 0.002], and the association appeared at least as strong as that between CS and MHDD.

### Correlation Between Contrast Sensitivity and Visual Acuity

As shown in Figure 3, there was good correlation between low contrast VA on the one hand, and both static [*r*_22_ = 0.74, *P* < 0.001] and dynamic [*r*_22_ = 0.78, *P* < 0.001] CS. This is consistent with the foregoing results and suggests that these measures index similar aspects of visual function.

### Correlation Between Contrast Sensitivity as Measured Clinically and Within the Driving Simulator

As shown in Figure 4, good correlations were observed between CS measured inside the simulator and via the clinical Optovist instrument. The correlation was particularly strong between the static-no-glare simulator and the no-glare Optovist measurements [*r*_13_ = 0.92, *P* < 0.001]. There was also a correlation between corresponding CS measurements made in the presence of glare [*r*_11_ = 0.69, *P* = 0.008], though this relationship appeared somewhat weaker, possibly owing to differences in the glare light source between the two instruments. Note also that across all conditions, CS was consistently estimated as being higher (better) in the driving simulator than that clinical Optovist device. It may be that this simply represents procedural differences in how the two psychophysical algorithms operated (Optovist determines the 60% correct threshold, whereas the BestPEST algorithm used in the simulator determines the 50% correct threshold). However, the exact reason for the difference was not investigated, and it is possible that there were also other, unknown factors at play.

### Effects of Glare, Motion, and Age on Contrast Sensitivity

Aggregating across both the dynamic and static viewing conditions, adding glare decreased CS in 20 of 22 (91%) of instances, with a mean difference of −0.29 logCS: A mean relative reduction of 41%. (Note *N* = 22, not *N* = 30, since not every participant completed every condition. See Table 1 for a detailed breakdown.) Moving from static to dynamic viewing decreased CS in 18 of 24 (75%) of instances, with a mean difference of −0.10 logCS: A mean relative reduction of 19% (again, see Table 1).

**TABLE 1:** Simple main effects of glare or motion on contrast sensitivity (CS).

	N data points	N (%) decreased	Mean Δ *logCS*
**Effect of adding glare:**			
Static viewing	13	12(92%)	−0.31
Dynamic viewing	9	8(89%)	−0.26
All	22	20(91%)	−0.29
**Effect of dynamic viewing:**			
No glare	15	11(73%)	−0.08
Glare	9	7(78%)	−0.14
All	24	18(75%)	−0.10

*For example, under dynamic viewing conditions the addition of glare caused logCS to decrease in 8 of 9 participants, with a mean reduction (deterioration) of 0.26 logCS. Note that N data points < 15 in many conditions due to missing data (not every participant completed every condition—see section “Materials and Methods”).*

To formally assess these effects statistically, a repeated measures two way ANOVA was conducted with independent variables of “glare” (two levels: no glare, glare) and “driving condition” (two levels: static, dynamic), and a dependent variable of logCS. For this analysis only those nine participants who completed every condition were included. This yielded significant main effects of glare [*F*_(1,8)_ = 22.47, *P* = 0.002] and driving condition [*F*_(1,8)_ = 11.45, *P* = 0.001], confirming that both factors affected CS (see Figure 5). No significant interaction was observed between these two factors [*F*_(1,8)_ = 0.03, *P* = 0.874].

As shown in Figure 6, mean CS, averaged across all four conditions, was also observed to deteriorate as a function of age [*r*_13_ = −0.72, *P* = 0.002], at a rate of approximately 0.45 logCS per decade.

## Discussion

Access to transport by car is crucial for the wellbeing and independence of many older people, and driving cessation is associated with increased levels of depression (Ragland et al., 2005), sedentary behaviors (Marottoli et al., 2000), and entry into long-term care (Freeman et al., 2006). However, as a society it is important that we are able to accurately and equitably evaluate fitness to drive, and given known biological changes in the aging eye (see Section “Introduction”) there have been longstanding calls for an unambiguous indicator of night driving ability for use with older adults (Sturgis and Osgood, 1982; Sturr et al., 1990; Gruber et al., 2013; Wood, 2020).

In the present study we demonstrate that contrast sensitivity (CS) in the healthy eye deteriorates between 50 and 80 years old (at a rate of ∼0.45 logCS/decade), and that these natural variations in CS are associated with poorer hazard detection performance within a night driving simulator. This relationship between CS and hazard detection performance was observed both with and without the presence of veiling glare (from simulated oncoming car headlights), though as would be expected CS was lower in the presence of glare. CS was also shown to decrease under “dynamic” testing conditions, in which the participant was simultaneously required to maintain a constant speed within the driving simulator. The data also showed that CS can be measured directly within a night driving simulator (providing results that were highly correlated with those from a standard clinical device: Optovist I), and that low contrast visual acuity was also associated with hazard detection performance, and was closely correlated with CS—suggesting that either may be an equally valid index of night driving ability.

### Comparisons With Previous Findings

Previous data from on-road studies indicate that photopic, high contrast VA, despite being the predominant measure of visual function when assessing fitness to drive, is a poor predictor of night driving ability (Anderson and Holliday, 1995; Gruber et al., 2013). Instead, Pelli Robson contrast sensitivity (Wood and Owens, 2005) and/or visual acuity measured under low illumination (Kimlin et al., 2017) have been suggested as better predictors of key night-driving metrics, such as obstacle recognition ability. Likewise, it has been shown that visual acuity alone is a poor predictor of night driving *cessation*, whereas older individuals with reduced CS are more likely to have limited their night driving activities (Kaleem et al., 2012). The findings of the present study are in good agreement with all of the above, with our data likewise indicating that high contrast VA does not well predict night-time hazard recognition ability, whereas CS and low contrast VA do.

### Implications and Study Limitations

The present study confirms that CS and/or low contrast acuity are able to capture variations in night-time hazard detection ability—abilities that are believed to decline markedly with age. These findings are consistent with previous calls from others that older drivers should pass a CS or low luminance VA examination in order to be eligible to drive at night (Sturgis and Osgood, 1982; Sturr et al., 1990; Wood, 2020). It is important to note, however, that the present findings do not directly speak to the real-world utility of such examinations, for the following reasons.

First, in the present study we only measured one component of night driving ability (hazard detection speed). In future, it would be highly beneficial to further consider the wide range of other driving metrics that have also been developed, such as those relating to speed control, braking, steering wheel control, lane keeping/changing, vehicle positioning, situational awareness, eye and head positioning, and so forth [e.g., see Östlund et al. (2005)]. Many of these measures could be well-studied within a simulated environment, though for a complete understanding of driving ability it is of course necessary to also look at convergent data from real world sources also (e.g., on-road surveillance, and actuarial data).

Second, it is possible that the deficit in hazard detection ability that we observed can be mitigated in the real-world by compensatory behaviors (e.g., more cautious driving or increased vigilance—or, in extremis, by night-driving cessation; see below). Against this stands a body of actuarial data that clearly shows elevated road-traffic crash and fatality rates amongst drivers over the age of 70 years (Mortimer and Fell, 1989; Cerrelli, 1998) (after correcting for yearly-mileage), strongly suggesting that at least some age-related deteriorations in driving ability cannot be effectively militated against by better driving strategies. It is important to note though that these age-related increases in accident rates are not necessarily the result of decreased visual function alone, and that cognitive and motoric deficits may also play at least as important a role (Ball and Owsley, 1991).

Third, for the routine evaluation of night-driving vision metrics to be useful, they would have to lead to a change in driving behaviors (e.g., a reduction or cessation of night driving). In this respect, it is important to note that 20–50% of older drivers already report having restricted or ceased night driving (Lyman et al., 2001; Naumann et al., 2011), with people exhibiting reduced CS being particularly likely to have done so (Kaleem et al., 2012). This could be taken to suggest that unlike, for example, visual field loss (Boodhna and Crabb, 2015), older drivers are well capable of noticing their loss of night time visual function, and of self-limiting their driving accordingly. Though whether all older drivers are so willing and able to do so, and the extent to which these changes to driving behavior are being made in a timely manner (i.e., prior to any road traffic incidents), is not known precisely, and remains a topic of longstanding investigation and debate (von Hebenstreit, 1984).

In short, while suggestive, the present data do not directly speak to the *utility* of routinely evaluating night vision metrics in older drivers. To conclusively demonstrate that such a program is a good use of time and resources, it would be necessary to prospectively evidence a real-world reduction in harms. Nonetheless, the present data do unequivocally suggest that such measures are worthy of further, serious consideration, particular given the fact that numbers of older drivers are increasing rapidly, due to both demographics (Vollset et al., 2020), and an increasing reliance of private car journeys in older people (Mollenkopf et al., 2002).

Aside from the issue of assessing fitness to drive, this work showcases more generally how next-generation night driving simulators, with motorized LED light sources to accurately simulate glare, can be used to help understand and evaluate the real world challenges that older road users may face. And to do so in a safe and standardized manner. This is not to suggest that simulators should be seen as superior to studies of on-road driving, which themselves have various unique advantages. Rather it is our belief that the two approaches should be seen as complementary, with simulators providing the highest degree of standardization, and convenience, as well as the ability to explore dangerous scenarios, while on-road studies provide richer and more unpredictable environments that may be more engaging for drivers, and which may place more stringent demands on their visuomotor abilities.

Finally, the present study may also have implications for the future design of vehicles and road traffic infrastructure. Thus, one particularly exciting aspect of night-driving simulations [i.e., as opposed to closed circuit tracks that have been predominantly used previously to assess night driving (Wood and Owens, 2005; Kimlin et al., 2017)] is that they permit engineering designs to be evaluated before they are built: after which point they often become prohibitively costly to alter. The present findings are encouraging, in that we able to demonstrate that the next generation of night driving simulators are sensitive to known changes in CS with age, and able to reproduce known associations between CS and aspects of night driving ability, in a safe and standardized manner. By extending the present set-up to introduce other sources of high-luminance light, such as streetlights, road signs, billboards, and building exterior lighting (i.e., in addition to oncoming car headlights), night-driving stimulators may in future offer an ideal testbed for engineers to evaluate the safety and efficacy of new, inclusive transport infrastructure, optimized for our increasingly aging societies.

## Data Availability Statement

Upon request, the raw data supporting the conclusions of this article will be made available by the authors, without undue reservation.

## Ethics Statement

The studies involving human participants were reviewed and approved by Institutional Review Board of the State Medical Association of Baden-Württemberg (F-2015-044#A2). The patients/participants provided their written informed consent to participate in this study.

## Author Contributions

JU, PE, MW, and US have contributed substantially to the conception and design of the work as well as to the acquisition of data. PJ, JU, MW, DC, and US have contributed substantially to the analysis and interpretation of data for the work, drafting the work, and revising it critically for important intellectual content. All authors provided approval for publication of the content and agreed to be accountable for all aspects of the work in ensuring that questions related to the accuracy or integrity of any part of the work were appropriately investigated and resolved.

## Conflict of Interest

JU had received a doctoral fellowship with funding from the Ministry of Science, Research and Arts Baden-Wuerttemberg as part of the HAW-Prom program. She was an employee at both Aalen University, Aalen/FRG and Carl Zeiss Vision International GmbH, Aalen/FRG. US was consultant of Haag-Streit AG, Köniz/CH. MW was Managing Director of Blickshift GmbH, Stuttgart/FRG. US and JU received a speakers’ honorary from AMO Ireland (affiliated to Johnson and Johnson Vision). US, MW, and JU were co-inventors for several patents/patent applications. The remaining authors declare that the research was conducted in the absence of any commercial or financial relationships that could be construed as a potential conflict of interest.

## Publisher’s Note

All claims expressed in this article are solely those of the authors and do not necessarily represent those of their affiliated organizations, or those of the publisher, the editors and the reviewers. Any product that may be evaluated in this article, or claim that may be made by its manufacturer, is not guaranteed or endorsed by the publisher.

## Supplementary Material

Supplementary Figure 1 CS measurements for the four conditions assessed in the driving simulator, shown for individual participants. The same data are expressed as group averaged in Figure 5 of the main manuscript.
