# Supplementary figures and images for: Contrast Sensitivity and Night Driving in Older People: Quantifying the Relationship Between Visual Acuity, Contrast Sensitivity, and Hazard Detection Distance in a Night-Time Driving Simulator
Source: Front Hum Neurosci. 2022 Jul 29;16:914459. doi: 10.3389/fnhum.2022.914459 (PMC9373794; doi:10.3389/fnhum.2022.914459)

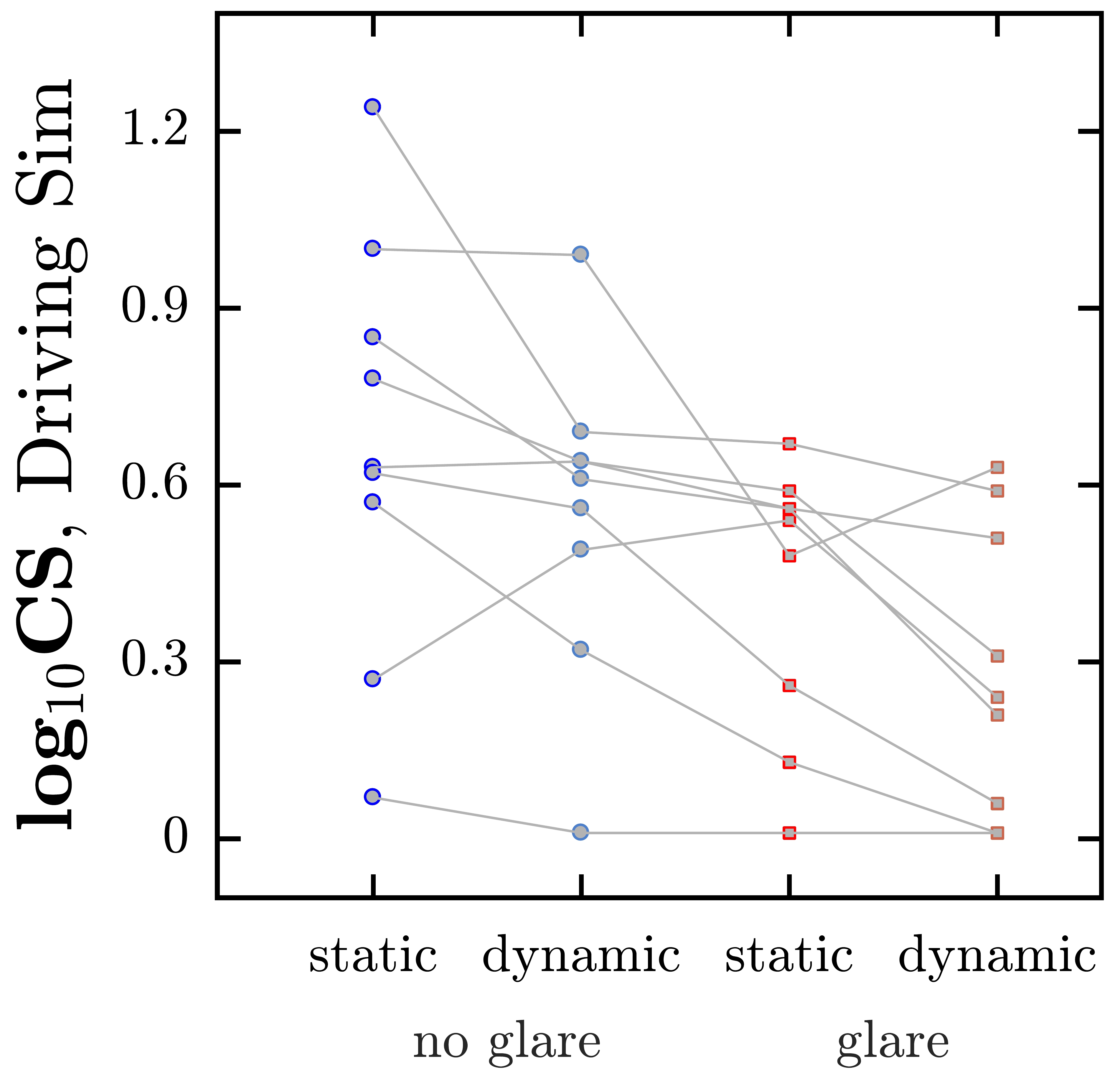

Supplement: Supplementary Figure 1 — CS measurements for the four conditions assessed in the driving simulator, shown for individual participants. The same data are expressed as group averaged in Figure 5 of the main manuscript. [file Image_1.JPEG]
